# High-Resolution Single Particle Zeta Potential Characterisation of Biological Nanoparticles using Tunable Resistive Pulse Sensing

**DOI:** 10.1038/s41598-017-14981-x

**Published:** 2017-12-12

**Authors:** Robert Vogel, Anoop K. Pal, Siddharth Jambhrunkar, Pragnesh Patel, Sachin S. Thakur, Eduardo Reátegui, Harendra S. Parekh, Paula Saá, Adonis Stassinopoulos, Murray F. Broom

**Affiliations:** 1 School of Mathematics and Physics, The University of Queensland, St Lucia, QLD 4072 Australia; 2 Izon Science US Limited, 85 Bolton Street, STE 108, Cambridge, MA 02140 USA; 3 Mucosal Diseases Group, Translational Research Institute, The University of Queensland, 37 Kent St., Woolloongabba, QLD 4102, Australia; 4 School of Pharmacy, The University of Queensland, 20 Cornwall St., Woolloongabba, QLD 4102 Australia; 5 William G. Lowrie Department of Chemical and Biomolecular Engineering, The Ohio State University, Columbus, OH 43210 USA; 6 The Comprehensive Cancer Center, The Ohio State University, Columbus, OH 43210 USA; 7 Scientific Affairs, American Red Cross, Rockville, MD 20877 USA; 8 Cerus Corporation, Concord, CA 94520 USA; 9 Izon Science Limited, 8C Homersham Place, PO Box 39168, Burnside, Christchurch 8053 New Zealand

## Abstract

Physicochemical properties of nanoparticles, such as size, shape, surface charge, density, and porosity play a central role in biological interactions and hence accurate determination of these characteristics is of utmost importance. Here we propose tunable resistive pulse sensing for simultaneous size and surface charge measurements on a particle-by-particle basis, enabling the analysis of a wide spectrum of nanoparticles and their mixtures. Existing methodologies for measuring zeta potential of nanoparticles using resistive pulse sensing are significantly improved by including convection into the theoretical model. The efficacy of this methodology is demonstrated for a range of biological case studies, including measurements of mixed anionic, cationic liposomes, extracellular vesicles in plasma, and *in situ* time study of DNA immobilisation on the surface of magnetic nanoparticles. The high-resolution single particle size and zeta potential characterisation will provide a better understanding of nano-bio interactions, positively impacting nanomedicine development and their regulatory approval.

## Introduction

Nanomedicine formulations typically represent a multifunctional and multicomponent system, making these vastly different from a small molecule drug^1,2^. This complexity is reflected in the diverse physicochemical properties possessed by a variety of nanomaterials, such as liposomes, extracellular vesicles, polymeric particles, micelles, metal colloids, and dendrimers, which are designed for diagnostic and therapeutic application in the pharmaceutical industry^1,2^. However, lack of substantial preclinical characterisation has been described as their “rate limiting step”, hampering their regulatory approval and commercialisation^1^. As the physicochemical properties of a nanoparticle influence its biological compatibility and thus govern the resulting outcome of nano-bio interactions^2,3^, a major emphasis is focused on accurate determination of key physicochemical particle properties, such as size, surface charge and hydrophobicity^3–5^. Hence, precise high-resolution nanoparticle characterisation plays a critical role not only in the nanomedicine formulation developmental process that includes fine tuning of nano-bio interactions^6^, but also during FDA regulatory submission, review and acceptance.

Zeta potential, which is indicative of the particle surface charge, is an important and widely used characterisation method of nanometer-sized objects in liquids, such as pharmaceuticals, inks, foams, liposomes and exosomes. The zeta potential represents the value of the electrostatic potential at the plane of shear and zeta potential values of typically ±30 mV are representative of stabilised particles^7^. Current characterisation methodologies are based on ensemble measurements (e.g. phase analysis light scattering, doppler velocimetry, streaming potentiometry) that measure the average electrophoretic mobility of particles in suspension. An ensemble approach becomes problematic when dealing with polydisperse samples that cover a wide zeta potential range.

Single particle electrokinetic measurements of nanoparticles using resistive pulse sensing were first shown by Deblois *et al*.^8^. Cylindrical nanopores, track-etched in polycarbonate were used to determine the electrophoretic mobility of latex spheres and various viruses. Particle velocities and respective electrophoretic mobilities were extracted from the translocation times of the particulates. Ito *et al*. used a comparable approach to determine the electrophoretic mobility of polystyrene nanoparticles using carbon-nanotube based pores^9–11^.

Recently it was shown that tunable resistive pulse sensing (TRPS), using conical thermoplastic polyurethane pores, is able to measure the zeta potential of individual particles based on the duration of the resistive pulse signal^12–15^. Arjmandi *et al*.^16^ applied a very similar approach, using solid state pyramidal shaped nanopores and measured the zeta potential of nanoparticles such as citrated gold particles and various types of viruses. The translocation duration of nanoparticles was measured as a function of voltage. From the inverse translocation time versus voltage dependency electrophoretic mobility and consecutively zeta potentials were calculated, which is comparable to the approach reported by Blundell *et al*.^15^ and Sikora *et al*.^12^.

Here we detail an advancement of resistive pulse sensing based methodologies (as described above) to allow for more robust and reproducible zeta potential measurements. The proposed modified method is based on measuring the translocation duration of nanoparticles as a function of both voltage and pressure. Compared with previous methods, convection is fully incorporated in the model in a more defined way. There exist numerous advantages of this convection inclusive approach compared with the methods described above, where electrophoresis dominates particle motion. These include the measurement of a wider spectrum of zeta potentials, the evaluation of very dilute particle suspensions and an increase in measurement resolution and accuracy. The unique capability of TRPS to simultaneously measure particle size and zeta potential on a particle-by-particle basis represents a new approach for investigating and understanding the heterogeneous properties of particle dispersions.

Herein we demonstrate the robustness and versatility of this improved methodology for different types of biologically relevant nanoparticles. These include measurements of multimodal mixtures of carboxylated and bare polystyrene particles, mixed anionic and cationic liposome samples, exosomes, and an *in situ* time study of DNA attachment onto magnetic nanoparticles.

## Results

### Methodology

The methodology for measuring zeta potential using TRPS has undergone an evolutionary process^13,15,17,18^, leading to a very robust and reproducible calibration-based zeta potential methodology. The method is based on the measurement of blockade event related signal durations as a function of both voltage and pressure. The average electrophoretic mobility of the calibration standard is calculated from the derivative of average particle velocity and applied electric field, whilst the convective flow is quantified through the derivative of average particle velocity and applied pressure. Zeta potentials of sample particles can then be calculated from respective electrophoretic mobilities using the Smoluchowski approximation^19^.

**Figure 1:**
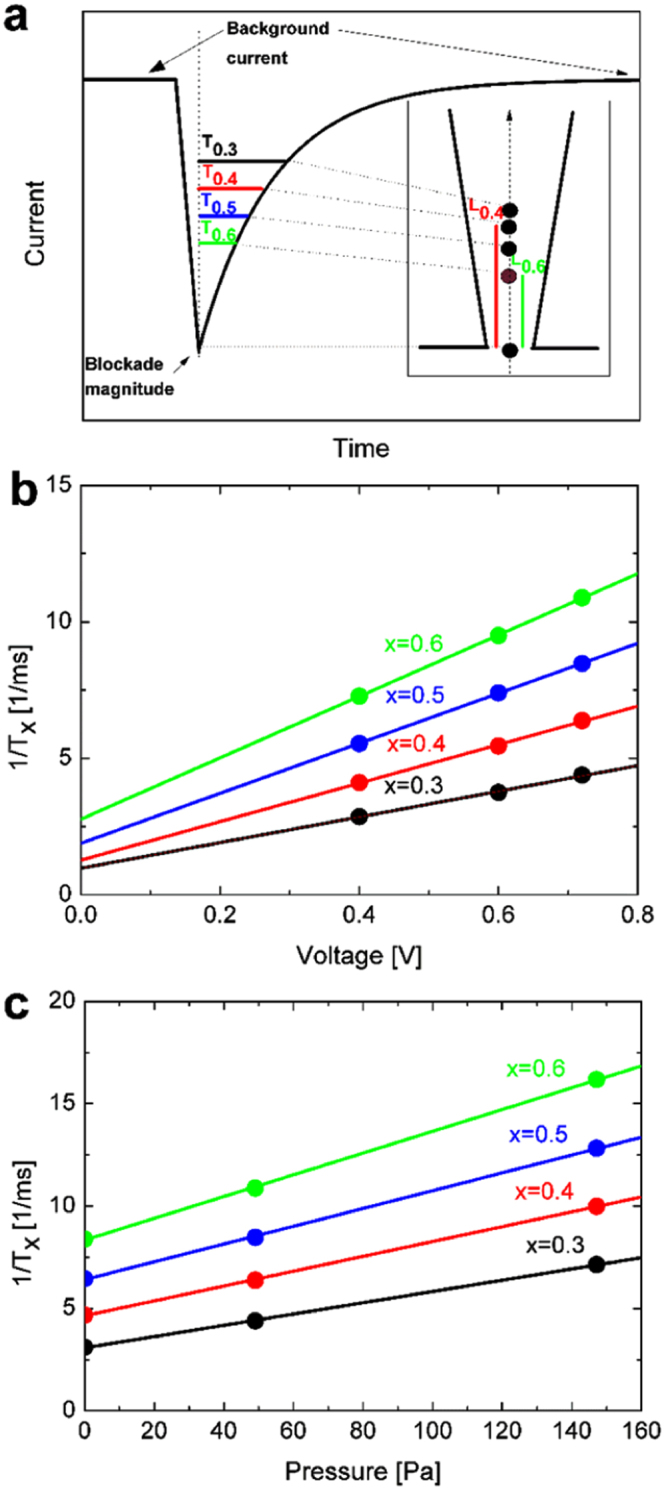
(**a**) A typical blockade event is generated as particles traverse a conical pore from the small to the large end. Various points of the translocation event are marked (*T*_*0.3*_, *T*_*0.4*_, *T*_*0.5*_, *T*_*0.6*_), representing a particle’s position (*L*_*0.3*_, *L*_*0.4*_, *L*_*0.5*_, *L*_*0.6*_) in the nanopore at any given time (see inset). *T*_1*.0*_ is the time the current blockade is at 100% magnitude, or in other words, the electric resistance is at its maximum. *T*_*0.4*_ and *T*_*0.6*_ are the times at which the blockade is 40% and 60% of the blockade maximum, respectively. (**b**) 1*/T* vs voltage linear curves used within the calibration method to measure differential electric mobilities. (**c**) *1/T* vs pressure linear curves used within the calibration method to quantify convection.

For each blockade, the time at which the blockade magnitude (peak) occurs is denoted as *T*_*1.0*_ (point of highest resistance in the signal trace). In the example shown in the inset of Fig. 1a, 4 different positions within the pore are indicated (*L*_*0.6*_, *L*_*0.5*_, *L*_*0.4*_ and *L*_*0.3*_), at which the signal trace reaches 60%, 50%, 40%, and 30% of the blockade magnitude, respectively (Fig. 1a). The time it takes from the occurrence of the blockade magnitude (*T*_*1.0*_) to each of these positions is defined as *T*_*0.6*_, *T*_*0.5*_, *T*_*0.4*_ and *T*_*0.3*,_ respectively. The pulse height at these sections, divided by the blockade magnitude is denoted with ‘proportional blockade magnitude’. Particles with different diameters are at the same position within the pore, when their proportional blockade magnitude is equal. Hence the proportional blockade magnitude can be used as a measure of particle position within the pore.

**Figure 4:**
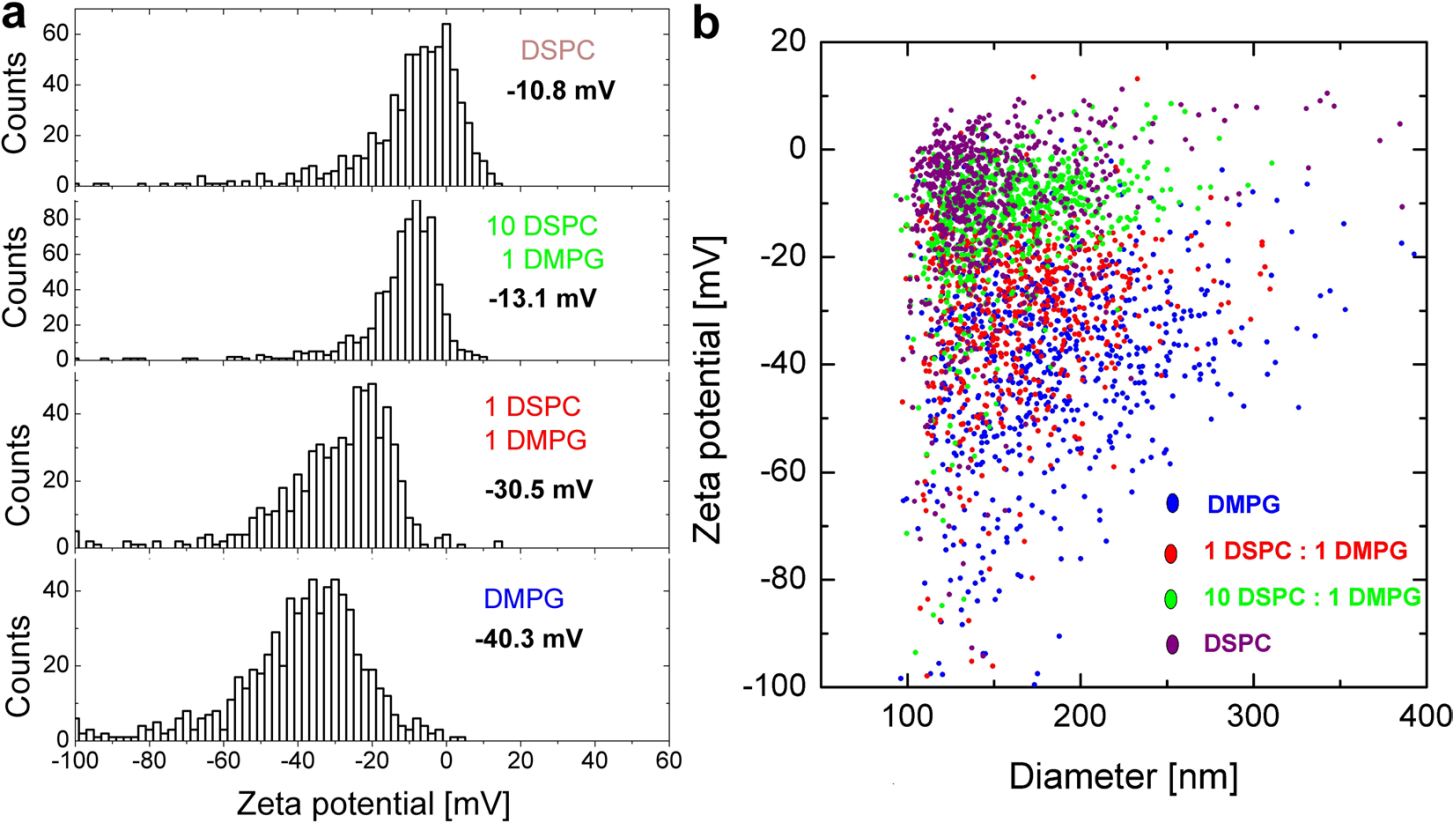
Simultaneous size and zeta potential measurements (**b**) of mixed DSPC + DMPG liposomes in phosphate buffered saline, using TRPS. DSPC and DMPG lipids were mixed in various molar ratios (1:0, 10:1, 1:1, 0:1) to form mixed liposomes and extruded with a 200 nm filter. The zeta potential of mixed liposomes becomes more negative with increasing DMPG content (**a**).

Pores are calibrated with standard carboxylated polystyrene particles with a known average zeta potential (Fig. 1b,c, Table S1), measuring the linear dependencies of *1*/*T*_*x*_ vs voltage, *V*, and *1/T*_*x*_ vs pressure, *P*. The step by step calibration process and the consecutive zeta potential calculation of the sample on a particle-by-particle basis were detailed in part in Blundell *et al*.^15^ and for reasons of completeness are listed again in the Supplementary Info (Equations A1–A10).

In brief, there is a linear relationship of electrokinetic (electroosmotic and electrophoretic) particle velocities of sample and calibration and respective zeta potentials (Equation 1), based on the Smoluchowski approximation^19^.

1 $$\frac{{({v}_{x}^{i})}_{elSample}\,}{{({v}_{x})}_{elCal}}=\frac{{\xi }_{x\,net\,Sample}^{i}}{{\xi }_{netCal}}$$

${({v}_{x}^{i})}_{{\rm{el}}{\rm{Sample}}}$ and ${({v}_{x})}_{{\rm{el}}{\rm{Cal}}}$ are time averaged electrokinetic velocities of sample particle, *i*, and calibration (averaged over at least 300 particles) at position *L*_*x*_ within the pore.

Net sample and calibration zeta potentials are the differences between respective particle zeta potentials and the membrane zeta potential, ${\xi }_{m}$.

2 $${\xi }_{pSample}\,={\xi }_{netSample}+{\xi }_{m}$$

The zeta potential of each sample particle, *i*, is

3 $${\xi }_{Sample}^{i}=\frac{{\sum }_{x}{\xi }_{x\,Sample}^{i}}{{{\rm{\Sigma }}}_{x}}=\frac{{\sum }_{x}({v}_{x\,Sample\,}^{i}-({v}_{x\,Cal}^{P}\,* \,{P}_{diff}+inte{r}_{xCal}))/({v}_{x\,Cal}^{V}\,* \,V)}{{{\rm{\Sigma }}}_{x}}* {\xi }_{netCal}+{\xi }_{m}$$

with *V*, *P*_*diff*_, *inter*_*xCal*_, ${v}_{x\,Cal}^{V}$, and ${v}_{x\,Cal}^{P}$ being the voltage used to run the sample, the difference of applied pressures between sample and calibration runs, the intercept and the slope of the *1/T*_*x*_ vs voltage linear curves, and the slope of the *1/T*_*x*_ vs pressure linear curves, respectively.

Evaluation at various positions within pore rather than just the end of sensing zone (as done in Arjmadi *et al*.^16^) is advantageous in that it enables effective quality control and elimination of rogue events such as electronic noise spikes. Blockade events with a variance in zeta potential (when averaged over various *T*_*x*_) larger than 9 mV^2^ were typically excluded from the analysis.

**Figure 2:**
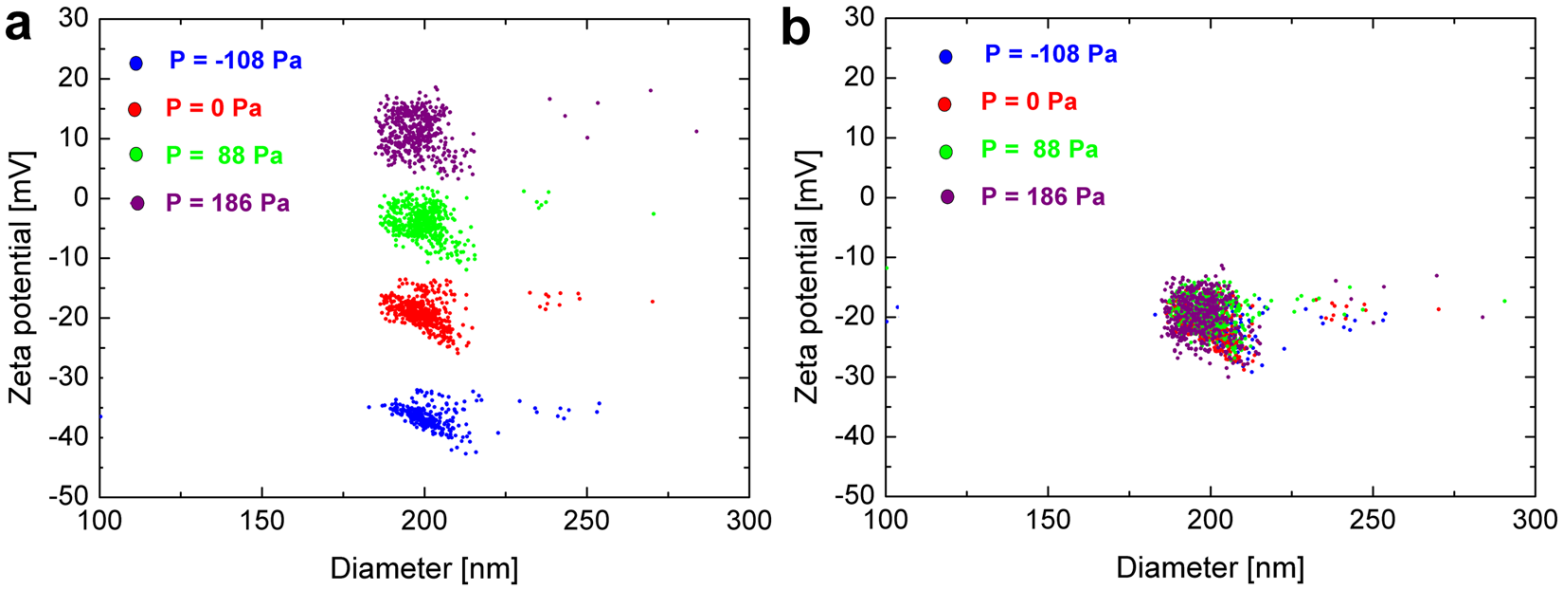
Comparison of zeta potential measurements for 200 nm carboxylated polystyrene particles CPC200 at various applied pressures. The results vary vastly with pressure when the convection term is estimated only by the intercept of the *1/T*_*x*_ vs voltage linear curves (**a**), whilst measured zeta potentials are independent of pressure when convection is well defined through the *1/T*_*x*_ vs pressure slopes (**b**).

Compared with previous methods, convection is fully included in a well-defined way. In Blundell *et al*. convective velocity was calculated from the intercept, whilst in the improved methodology described here, convection is defined by the *1*/*T* vs pressure slopes. The first approach is inherently ill-defined, as the intercept partially originates from pore asymmetry, and the energy/entropy barrier particles face to translocate the pore^16^. Hence this approach will only give accurate results for very small applied pressures where electrokinetics dominates particle motion, whilst the improved methodology shows very good stability over a range of applied pressures (see Fig. 2).

The present convective flow will affect total particle velocities. There are various advantages of this convection inclusive approach, compared with the methods described above. Capturing the whole spectrum of zeta potentials of samples with a very wide range of zeta potentials, in particular when reaching from positive to negative values, will require the application of an external pressure. The consideration of convection also becomes important for the evaluation of zeta potentials of very dilute particle suspensions, where a method purely based on electrokinetics becomes impractical due to low count rates. In another scenario, in the case of highly charged particles an opposed pressure might be required to slow the particles sufficiently down, to allow for a better resolution of the blockade shape for each particle.

### Stability of measurements at different applied pressures

The stability of zeta potential results for CPC200 at various applied pressures is demonstrated in Fig. 2. Whilst the zeta potential changes significantly with varying pressure (Fig. 2a) when the theoretical model predicts convection through the intercepts of the *1*/*T*_*x*_ vs voltage, results are very stable (−20.5 mV ± 1.0 mV) for various pressures reaching from −108 Pa to 186 Pa, when convection is well defined through the slopes of the *1*/*T*_*x*_ vs pressure linear curves (Fig. 2b).

### Zeta potential measurement reproducibility

Zeta potential measurements were shown to be stable, with CVs (=standard deviation/mean) smaller than 5% for a range of applied pressures (Fig. 2). CVs, when using different pores and stretches were approximately 3–8%, with the CV being highest for near neutral particles (Table S2, Fig. S1). Results were most repeatable when the same pore was used and samples were run in direct succession. This guaranteed that the thermoplastic polyurethane pores did not change its geometry in between samples. When CPC200 and CPN200 were measured repeatedly (>10x), whilst cleaning the fluid cell with electrolyte in between sample runs, CVs were only 1.6% and 0.9%, respectively.

### Multimodal particle populations

**Figure 3:**
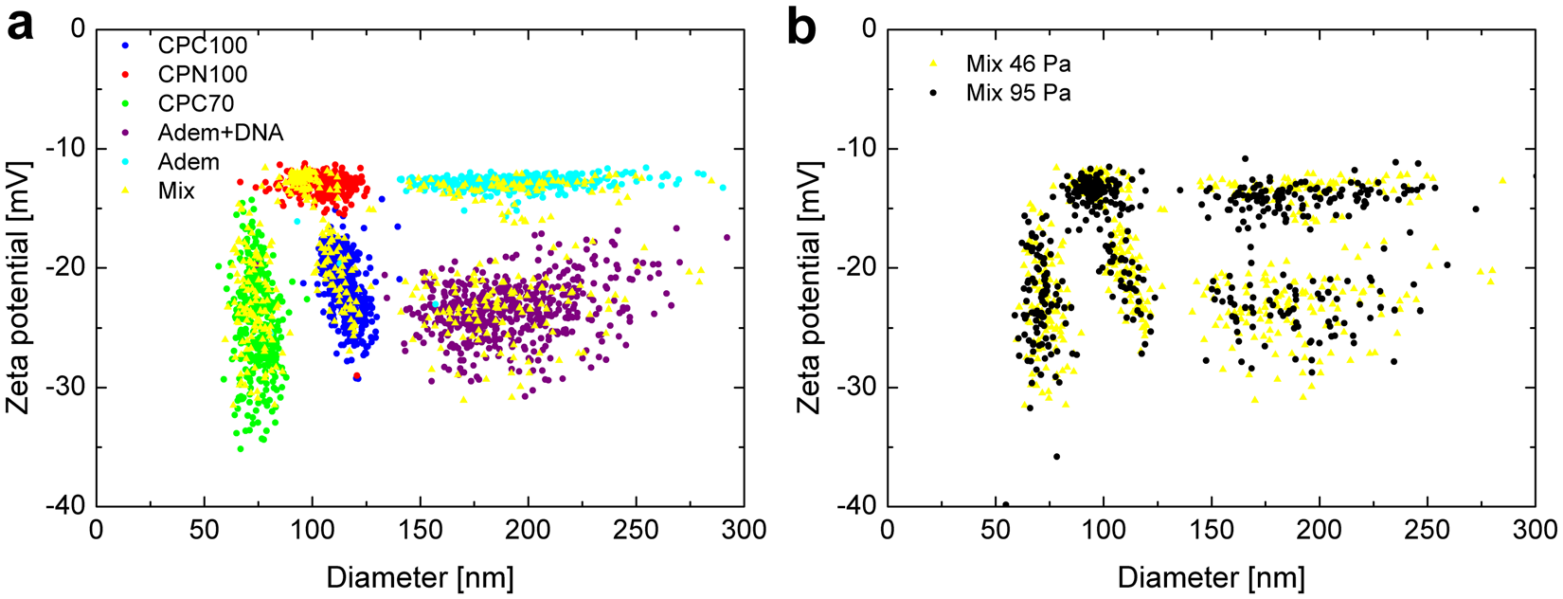
(**a**) Zeta potential vs particle size of bare polystyrene (CPN100), carboxylated polystyrene particles (CPC70, CPC100), magnetic particles (Bio-Adembeads) and magnetic particles modified with DNA. The mix of all 5 particle types (in yellow) resembles very well the particle distributions when particle types are measured separately. (**b**) There is good agreement of the penta-modal distribution when the mix is run at different pressures (46 and 95 Pa).

The single-particle nature of TRPS makes the discrimination of subpopulations within a sample possible. This applies not only to multimodal size distributions, but also to multimodal surface charge distributions (Fig. 3 and Fig. S3). A mixed sample of CPN380 and CPC400 with equivalent sizes but different zeta potentials could not be resolved with phase analysis light scattering (PALS), which is an ensemble technique (Fig. S3)^20^. However, the same sample, when analysed with TRPS, displayed clearly defined populations (Fig.S3)^20^. Ensemble techniques, as opposed to single particle measurement techniques (such as TRPS), only measure and calculate the average particle properties and hence detailed information is lost, in particular when measuring polydisperse samples.

In another example, zeta potential and particle size were simultaneously measured for a penta-modal mix of bare polystyrene (CPN100), carboxylated polystyrene particles (CPC70, CPC100), magnetic particles (Bio-Adembeads) and magnetic particles modified with DNA. The 5 particle types were fully resolved in size and zeta potential. Results from the mix agreed very well with results when particles were measured separately. There is also good agreement of the penta-modal distribution when the mix is run at different pressures (46 and 95 Pa), again confirming the stability and robustness of the described methodology.

The particle properties of CPN100, CPC70, CPC100, Bio-Adembeads, and Bio-Adembeads + DNA, as given by the particle providers are listed in Table 1 and compared with respective TRPS results. In terms of particle diameter and its spread, quantified with the coefficient of variance (CV), spec sheet values from particle providers agree very well with respective TRPS results.

**Table 1:** Mean diameter, CV, parking area and zeta potential as specified in data sheets (obtained from particle providers), and as measured with TRPS and PALS.

Particles	Mean Diameter (CV) *spec sheets*	Mean Diameter (CV) *TRPS*	Parking area *spec sheets*	Zeta potential *TRPS (PALS)*
CPC70	69 nm (5–10%)	74 nm (8.1%)	0.439 nm^2^	−24.5 mV (−18)
CPC100	120 nm ± 5 nm (5–10%)	115 nm (5.4%)	0.458 nm^2^	−21.3 mV (−21)
CPN100	100 nm ± 3 nm (7.8%)	102 nm (9.1%)	Not available	−12.9 mV (−12)
Bio Adem	200 nm ± 15 nm (<20%)	191 nm (14.6%)	Not available	−12.9 mV (N.av.)
Bio Adem + DNA	200 nm ± 15 nm (<20%)	192 nm (14.5%)	Not available	−23.5 mV (N.av.)

In terms of zeta potential measurements there is good agreement between TRPS and PALS measurements for CPN100 and CPC100. Bangs Labs specifies its carboxylated polystyrene particles (CPCs) with a parking area, which is the area per functional group. In the Smoluchowski limit for not too large absolute zeta potentials the particle zeta potential scales linearly with the surface charge density^19^, which is indirectly proportional to the charged functional group parking area. With COOH parking areas for CPC70 and CPC100 being comparable we also expect their zeta potentials to be comparable. However, the mean zeta potential of the CPC70 is marginally more negative than expected, when comparing its parking area and measured zeta potential with respective values for CPC100. The wide spread in zeta potential for CPC70 is possibly due to a combination of two factors: First, a variation in COOH coverage within the particle population and second, a combination of limited sampling frequency and CPC70 particles having very small blockades, approaching the electronic noise level.

Bare polystyrene particles (CPNs) have a small non-zero surface charge due to sulfate surface functionality. Carboxylation of bare polystyrene particles results in different degrees of COOH surface coverage within the particle population and hence an increase in spread of zeta potential, compared with bare polystyrene particles. In case of Bio-Adembeads surface functionalisation with DNA leads to a more negative average zeta potential and also an increased spread in measured zeta potential compared with unmodified Bio-Adembeads due to different degrees of DNA coverage within the particle population.

### Mixed liposomes

Liposomes are one of the most commercially established and regulatory accepted nanoparticles for drug delivery^14^. Mixed anionic, cationic liposomes, in particular, have been used to mimic cellular membranes^21^. Binding of proteins to plasma membranes, such as the heat shock protein Hsp 70 which is essential for maintaining homeostasis, was studied using mixed liposome formulations^22,23^. The surface charge of the liposome membrane will influence the electrostatic interaction with proteins, cell surfaces, etc., and hence may play an important role in understanding their binding process.

TRPS enables the number concentration and volume fraction of liposomes, as well as particle-by-particle size and zeta potential distribution to be determined^14^. Here we demonstrate the high resolution TRPS zeta potential measurement capability of TRPS on mixed DSPC (1,2-distearoyl-*sn*-glycero-3-phosphocholine) + DMPG (1,2-dimyristoyl-*sn*-glycero-3-phosphorylglycerol sodium salt) liposomes with various DSPC: DMPG molar ratios (Fig. 4). PC and PG based phospholipids are both present in eukaryotic plasma membranes, with PC being the major component and hence this model system might have  utility for protein binding studies.

Many liposome properties depend on the characteristics of the bilayer solution interface^24^. The charge and the polarity of the lipid head groups strongly affect the conformation and phase behaviour, in particular, the phase transition temperatures of liposomes^25,26^. Similar phase behaviour is important to consider when mixing different lipids. For example, PC and PG, and also PE and PS based lipids show nearly perfect miscibility^27^, whilst PC and PE based lipids are only weakly miscible. Avanti Lipids predicts medium miscibility for DSPC and DMPG due to the difference in fatty acid chain length^28^.

A simple means of tracking the successful mixing of different lipids into mixed liposomes is via a change in their electrophoretic mobility (zeta potential), which arises from the change in the number of charged external surface groups. PC lipids are zwitterionic, that is, each molecule possesses an equal number of positive and negatively charged groups, and therefore when assembled into a liposome they have no net surface charge. The measured zeta potential for pure DSPC liposomes in phosphate buffered saline (PBS) was −10.8 ± 1.5 mV (Fig. 4). PG lipids are negatively charged and therefore when assembled into a liposome they acquire a negative charge. The measured zeta potential for pure DMPG liposomes in PBS was −40.3 ± 4.3 mV (Fig. 4). When used to form a mixed liposome, the number or ratio of charged to uncharged lipids can be detected as a difference in the liposome zeta potential. For example, the number or ratio of PG to PC phospholipids incorporated into the liposome, can be monitored from the corresponding decrease (becoming more negative) in the zeta potential of the particles (Fig. 4).

The TRPS zeta potential results were compared with PALS results. Measured PALS zeta potentials (triplicates) for pure DSPC, 10:1 DSPC:DMPG, 1:1 DSPC:DMPG and pure DMPG were −9.4 ± 0.5 mV, −15.0 ± 1.8 mV, −32.8 ± 1.3 mV, −38.4 ± 1.1 mV respectively, being in very good agreement with TRPS results.

### Extracellular Vesicle (EV) samples

Extracellular vesicles or EVs are small membrane-bound vesicles that are released by cells and can be found circulating in biological fluids, including blood^29,30^. EVs contain “cargo” including nucleic acids and proteins, which vary depending on pathological and physiological conditions^31,32^. Following their release from the cell, EVs can fuse and transfer their cargo into target cells where biological events may be triggered^32^. EVs have been involved in important biological and pathological processes such as cell/tissue differentiation, antigen presentation^33,34^, angiogenesis^35^, coagulation and inflammation^36^, among others. Within the context of infectious diseases, EVs have been found to contain host and pathogen-derived molecules that may induce immune responses by promoting cytokine production and facilitate antigen presentation^37^.

New strategies have been developed to remove or inactivate infectious agents and lymphocytes in blood and its derivatives, one of them being Cerus’ INTERCEPT Blood System for plasma and platelets. This technology combines amotosalen with ultraviolet (UVA) illumination to specifically target nucleic acids and irreversibly cross-link or form adducts on these molecules, thereby blocking pathogen replication. The efficacy and safety of amotosalen/UVA treated platelet and plasma components has been extensively investigated, however the effects on EVs and their biological activity have not been extensively explored.

**Figure 5:**
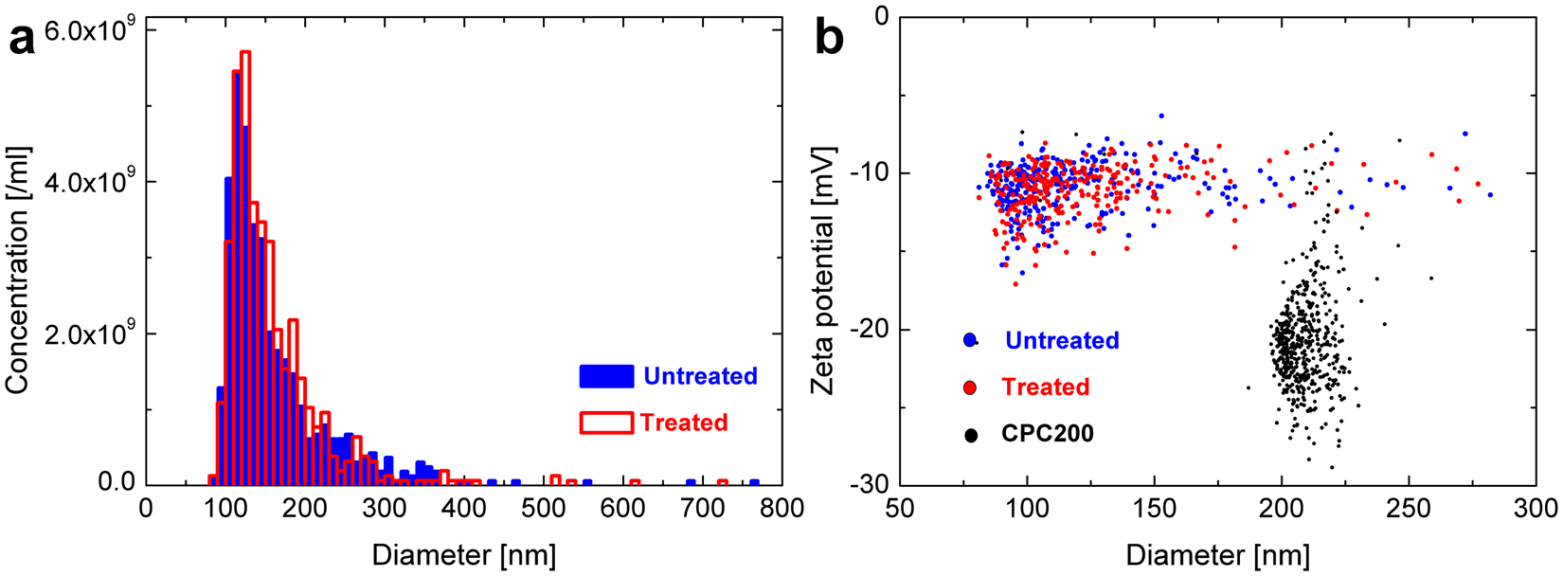
(**a**) Particle concentration vs. size measurements for EV samples before and after INTERCEPT treatment show no major difference. (**b**) Simultaneous size and zeta potential measurements of untreated, INTERCEPT treated EV samples, and carboxylated polystyrene standard particles.

Detailed measurements (see Fig. 5a and Table S4) using TRPS of pooled size exclusion column fractions 9–11 showed the absence of statistically significant differences in average EV concentration and size. Average concentrations within the size range between 80 nm and 1000 nm, C_80-1000,_ were 3.4*10^10^/ml vs. 3.8*10^10^/ml and average EV diameters 157 nm vs. 158 nm between pre- and post-treated samples of 6 healthy volunteer donors, respectively. Likewise, zeta potential analyses of these fractions (Fig. 5b) found no changes in extra cellular vesicle zeta potential following INTECTEPT treatment (−10.9 mV vs. −11.1 mV). The TRPS zeta potential results were compared with PALS results, which were −12.4 ± 0.5 mV and −12.4 ± 0.4 mV, respectively.

Also, preliminary protein analysis of EV lysates by quantitative and qualitative assays did not show major phenotypic variations, and TEM imaging did not indicate any qualitative changes between treated and untreated samples (results not shown).

### DNA-time study

The immobilisation of oligonucleotides onto surfaces has been crucial to many applications within DNA sequencing^38,39^, DNA-protein interactions^40–42^, biosensing^43–45^ and targeted drug delivery^9–11^. Particle-based assays often use magnetic particles due to enhanced purification and removal of specific analytes from complex sample matrices, e.g. using magnetic devices^46–50^. Combined with fluidic systems magnetic particles can be used for DNA and cell sorting^51^, and a variety of detection platforms^46,51,52^.

**Figure 6:**
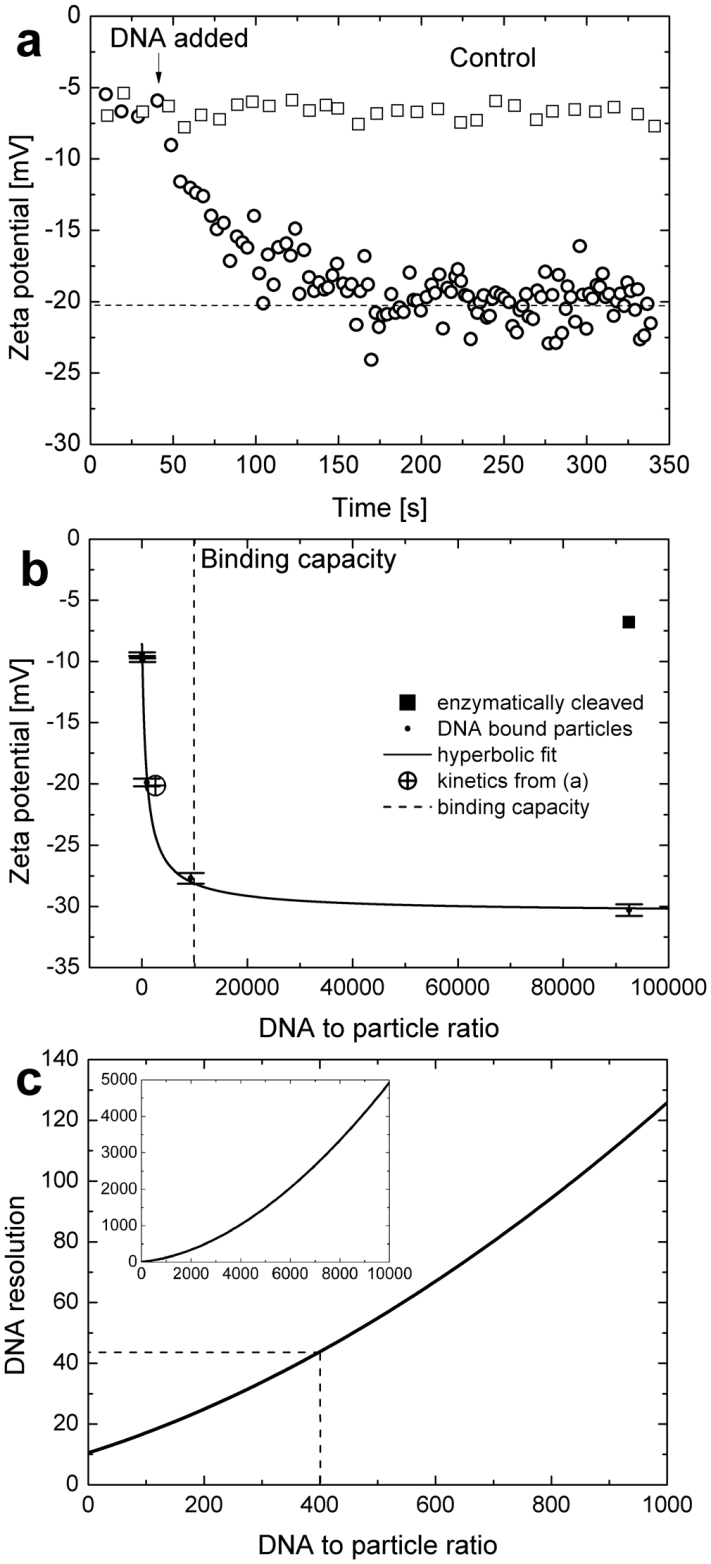
Kinetics of biotinylated DNA reaction onto Bio-Adembeads was monitored via particle zeta potential measurements (**a**) and compared with a control where streptavidin on the surface of the Bio-Adembeads was enzymatically cleaved before DNA reaction. Approximately 100 sec after DNA addition to the fluid cell the moving average of the zeta potential (averaged over 25 particles) levelled at approximately −20 mV, indicating reaction saturation. The zeta potential for the control does not change with time. The average zeta potential at saturation for various DNA to particle ratios follows a hyperbolic behaviour (**b**). The saturation zeta potential for the specific case in (**a**) is marked with a circled cross. Enzymatically cleaved Bio-Adembeads (marked with a square) showed no change in zeta potential after incubation with DNA, indicating that the reaction is highly specific. The binding capacity of the streptavidin modified particles as stated by the particle provider is indicated by the dashed line. Error bars for DNA bound particles are shown and for the kinetic and enzymatically cleaved samples indicated by the size of the marker. The resolution, defined as the number of DNA molecules per particle which can be distinguished via zeta potential measurements is plotted in (**c**). The inset shows the expanded view. For example, in the case of the dashed line in (**c**) for a loading of 400 DNA molecules per particle, the resolution is approximately 44 DNA molecules.

Fig. 6a shows the kinetics of biotinylated DNA (35mer) reaction onto streptavidin modified magnetic particles which was successfully monitored via particle zeta potential measurements. Starting from approximately −7 mV the moving average of the zeta potential (averaged over 25 particles) increased in absolute value post addition of DNA to the top fluid cell (which contains the sample particles) and 100 seconds later levelled at approximately −20 mV, indicating reaction saturation. This was compared with a control where streptavidin on the Bio-Adembead surface was enzymatically cleaved before DNA addition. In this case the zeta potential remained constant at approximately −7 mV post DNA addition. This demonstrates that the reaction is highly specific.

Pores were lined with Izon pore coating solution (ICS) to prevent non-specific binding of DNA to the pore surface. Details of pore coating procedure and its effect on zeta potential measurements are discussed in SI (Table S3 and Fig. S2).

In order to study the relationship of particle zeta potential and the DNA per particle ratio, Bio-Adembeads were incubated at various DNA concentrations (with DNA/particle ratios ranging from 9.25–92500) and reactions brought to completion, before measuring the reacted particles using TRPS. Fig. 6b demonstrates that the average zeta potential at saturation for various DNA to particle ratios follows a hyperbolic behaviour. The saturation zeta potential for the specific case in (a) is marked with a circled cross. Enzymatically cleaved Bio-Adembeads (marked with a square) showed no change in zeta potential after incubation with DNA, indicating that the reaction is highly specific.

The binding capacity of the streptavidin modified particles as stated by the particle provider is indicated by the dashed line. Error bars for DNA bound particles are shown and for the kinetic and enzymatically cleaved samples indicated by the size of the marker. Due to the high affinity of streptavidin for biotinylated DNA, all the DNA is rapidly bound to the particles when the DNA concentration is limiting relative to available streptavidin.

For best relative and most comparative zeta potential results between samples, it is best practice to run samples in direct succession using the same pore at the same stretch^15,53^. As discussed earlier in the ‘zeta potential measurement reproducibility’ section the coefficient of variance of averaged zeta potentials of CPC200 was less than 1.6% and less than 1% for CPN200, when sample runs were repeated in direct succession using the same pore. In comparison, CVs were approximately 4.5% and 8% respectively, when different pores were used. Please note, that control and the kinetic experiments (square and circled cross in Fig. 6b) were both collected, using a different pore from the samples constituting the hyperbolic curve and hence zeta potentials deviated slightly from the expected values, defined by the hyperbolic curve.

Assuming a CV of 1.6% for DNA reacted Bio-Adembeads from Fig. 6b the measurable difference in DNA particle coverage via zeta potential measurements is plotted in Fig. 6c. This curve was generated from differentiating the hyperbolic curve and assuming that zeta potentials with a difference of 2 standard deviations (2 × 1.6%) can be distinguished with at least 95% certainty (given that mean zeta potentials are normally distributed). The resolution, defined as the number of DNA molecules per particles which can be distinguished via zeta potential measurement decreases with increasing DNA loading. For example, in case of the dashed line in Fig. 6c for a loading of 400 DNA molecules per particle, the resolution is approximately 44 DNA molecules. This means that particles with an average loading of 378 can be distinguished with more than 95% percent certainty from particles with an average loading of 422. This resolution will possibly be higher when longer, more highly charged DNA molecules were to be used instead of the 35mers (see Materials and Methods).

## Discussion

Policy makers and regulatory agencies around the world have rightly emphasised the need  to include particle size, size distribution, surface charge and particle concentration in nanotechnology-based product submissions for scientific regulatory review and approval^20,54^. For instance, particle surface charge measurement (zeta potential) can provide useful surrogate information about the nanoparticle fate and nano-bio interaction in different biological media of choice^3,55^. Currently, several particle characterisation techniques (such as TRPS, DLS/PALS, TEM, AFM, NTA, flow cytometry, etc.) exist, of which only TRPS provides simultaneous in-suspension information about particle size and zeta potential on particle-by-particle basis^13,15^.

In this study, we have provided a convincing case for improved particle characterisation through TRPS methodology, leading to accurate determination of particle zeta potential of different biologically relevant nanoparticles. In addition, this technique has been shown to provide high-resolution size and precise particle concentration analysis over a defined size range. Advantages of the described size and zeta potential measurement methodology over previously reported methodologies include analysis of wider spectrum of zeta potentials, higher measurement precision and accuracy, and the analysis of dilute solutions.

Different case studies were designed to demonstrate the utility of this improved methodology with regards to nanoparticles, routinely explored for drug-delivery and therapeutic applications. Mixed phospholipid liposome formulations of weakly charged DSPC and highly charged DMPG, using various molar ratios clearly could be distinguished due to the difference in mean zeta potential and its distribution. Human plasma extracellular vesicle samples were measured with TRPS before and after INTERCEPT blood treatment, which is used to neutralise pathogens. As predicted, size distribution, concentration and zeta potential of the extracellular vesicles were shown to remain unchanged, with TRPS and PALS zeta potential measurements being in very good agreement (difference <1.5 mV). Finally, the *in-situ* immobilisation of DNA on magnetic particles was monitored via zeta potential measurements. After DNA addition to the magnetic particles, the rolling average of the measured particle zeta potential became more negative and quickly plateaued, indicating that reaction had reached completion (saturation). A calibration curve of the relationship between mean zeta potential at saturation and the DNA to particle ratio can be used to predict the DNA coverage of an unknown sample with high accuracy.

The presented TRPS measurement zeta potential methodology can be readily applied to any nano-bio particulate system, in which the particles are dispersed in aqueous electrolyte solutions.

## Materials and Methods

### Polystyrene particles

Carboxylated polystyrene particle standards with nominal diameters of approximately 70 nm, 100 nm, 200 nm, 350 nm, and 400 nm were purchased from Bangs Laboratories and Polysciences. Bare, un-carboxylated polystyrene particles (NIST traceable size standards) with nominal diameters of approximately 100 nm, 200 nm, and 380 nm were purchased from Polysciences. Carboxylated particles are denoted as CPC and un-carboxylated particles as CPN.

### Magnetic particles

Streptavidin modified magnetic particles with a nominal diameter of 200 ± 15 nm were obtained from Ademtech France (Bio-Adembeads Streptavidin Plus 0322). The particle’s binding capacity is 1960 pmoles biotin/mg. For binding studies, a 50 µl aliquot of Bio-Adembeads was re-suspended in freshly prepared and filtered electrolyte (PBS, 0.03% Tween, 2% Izon Coating Solution), magnetically separated for 4 minutes, washed × 3 with 200 µl of electrolyte and re-suspended in above electrolyte, denoted as ‘working-electrolyte’. The concentration of washed particles was determined with TRPS.

### DNA modification of Bio-Adembeads

The random DNA 35mer (5′-AATGTTAGTTTGCTGGTTCATGTAAAAATTCTTTA-3′) with a terminal 3′ biotin. was obtained from GeneWorks Pty Ltd Australia. Frozen stocks of the oligo were stored at 10 pM/µl in MilliQ water with 0.5 mM EDTA.

For monitoring real-time DNA to particle binding via TRPS, washed Bio-Adembeads were re-suspended in the electrolyte at a concentration of approximately 6.5 × 10^9^ particles/ml (as determined with TRPS) and 35 µl added to the qNano upper fluid cell. After approximately 40 sec of recording blockades with TRPS an aliquot of 1 µl biotinylated DNA oligo (1 pM/µl) was added to the upper fluid cell and the change in blockade duration, indicative of DNA binding monitored in time.

As control the above kinetic experiment was repeated with Bio-Adembeads with inactivated streptavidin. Bio-Adembeads (10 µl) were inactivated by incubation in a working-electrolyte solution of proteinase K (10 µg/µl) at 37 °C for 1.5 hr. Before TRPS measurement these particles were magnetically separated, washed and re-suspended in electrolyte (PBS, 0.03% Tween 20, 2% ICS).

In order to study the relationship between zeta potential of DNA modified Bio-Adembeads and the DNA to particle ratio, beads (6.51 × 10^8^) were incubated in 100 µl of working-electrolyte with various DNA concentrations (10 pmol/µl, 1 pmol/µl, 10^−1^ pmol/µl, 10^−2^ pmol/µl, 10^−3^ pmol/µl, and 10^−4^ pmol/µl) for 1 hr at 37 °C. After completed incubation, particle zeta potential and diameter for at least 300 particles/sample were measured with TRPS. This measurement was repeated for control reactions that used enzymatically (proteinase K) cleaved Bio-Adembeads (at 1 pmol/µl).

### Mixed liposome preparation

Liposomes were prepared, using zwitterionic 1,2-distearoyl-sn-glycero-3-phosphocholine (DSPC) and negatively charged 1,2-dimyristoyl-sn-glycero-3-phosphorylglycerol sodium salt (DMPG). The phospholipids were bought from Avanti Polar Lipids. Liposomes were prepared using a thin film hydration method which yielded large multilamellar vesicles that were subjected to an extrusion process to obtain small unilamellar vesicles. Liposomes of different surface charge were prepared by combining DSPC and DMPG at various molar ratios (1:1, 0:1, 1:0 and 10:1).

The phospholipids as per combination mentioned above were weighed in a rotary evaporator (B$\ddot{u}$chi Labortechnik AG) flask followed by addition of 5 ml of ethanol. The rotary evaporator flask containing ethanolic dispersion was allowed to mix thoroughly at 60–65 °C to obtain a clear solution, before the ethanol was evaporated under reduced vacuum for 20 minutes at 20 rpm. Once a layer of thin film formed on the wall of the flask, residual ethanol was removed by passing a stream of nitrogen gas over the film. Finally, 10 ml of PBS solution was pipetted into the flask and the content was allowed to mix by rotation at 100 rpm at 65 °C for 15 minutes. This liposomal formulation was subjected to a series of extrusions (five times per membrane) through Whatman polycarbonate membranes (GE Healthcare Australia) of pore sizes 800 nm, 400 nm, and 200 nm using a LIPEX^TM^ extruder (Northern Lipids Inc.) to obtain liposomes of 200 nm size. Finally, the formulations were characterised with TRPS (IZON Science Ltd.) and Zetasizer Nano ZS (Malvern Instruments Ltd.).

### Extracellular vesicles

For this extracellular vesicle case study, apheresis platelet concentrates (PC) in 100 percent plasma were collected from healthy donors and pathogen inactivated following standard procedures.

This study and related methods were conducted in accordance with the Declaration of Helsinki. All the experimental protocols for this study were reviewed and approved by American Red Cross Institutional Review Board. The IRB protocol number covering the experiments delineated in the manuscript is 2014–027. All donors received informed consent and agreed to participate in the study.

For inactivating infectious agents and lymphocytes in platelet concentrates Cerus’ INTERCEPT^TM^ blood system for platelets was used. INTERCEPT™ Blood System combines amotosalen addition to the apheresis concentrates with ultraviolet (UVA) illumination to irreversibly form adducts or cross-link nucleic acid molecules and subsequently inhibit pathogen and lymphocyte replication. Amotosalen is a light-activated, DNA-, RNA-reactive psoralen compound, which is used to inactivate pathogens.

To evaluate changes in EV population size distribution, concentration and surface charge, a control sample aliquot was removed from the PCs after amotosalen addition, but before UVA illumination, the latter causing nucleic acid cross-linking^56^. PC units were then illuminated and a post-illuminated sample aliquots collected. EVs were isolated from pre- and post-illuminated (also denoted as post-treated) samples by centrifugation and size exclusion chromatography (Exo‐Spin Midi Columns, Cell Guidance Systems, St Louis, MO). The presence of EVs and changes in size, concentration, and zeta potential were evaluated with antibody array, transmission electron microscopy (TEM), and TRPS, respectively.

### Nanopores and pore coating

For all experiments pertinent to this study thermoplastic polyurethane nanopores^57^ (Izon Science Ltd.) were used. For all DNA-related experiments nanopores were lined with Izon pore coating solution (ICS), which is a protein-free PBS-based formulation. ICS reduces non-specific binding and hence reduces the occurrence of blockages and unwanted pore-modifications^58^.

For experiments where no ICS coating was applied, the pores were wetted and equilibrated at maximum pressure with freshly prepared and filtered electrolyte (0.22micron) PBS containing Tween 20 (0.03% v/v). For preparing coated pores, the wetted pore (prepared as above) was coated under a pressure of approximately 2000 Pa for 30 minutes with filtered 10% Izon coating solution. After the coating procedure, the pore was re-equilibrated into electrolyte (freshly prepared PBS, 0.03% Tween 20, 2% Izon Coating solution), with the 2% Izon coating solution, maintaining a stable coating of the pore throughout the experiment.

### TRPS setup

All measurements were conducted using the qNano (Izon Science Ltd., NZ) and Izon Control Suite v.3.3. For all qNano experiments, the lower and upper fluid cells contained 80 µl of electrolyte buffer, whilst the upper fluid cell contained 35 µl of sample. A detailed description of qNano can be found in Willmott *et al*.^59^ and Vogel *et al*.^60^.

### Calibration of zeta potential measurements

Zeta potentials of carboxylated polystyrene calibration particles were measured by phase analysis light scattering (Malvern Zetasizer Nano ZS), using disposable cuvettes. Measured zeta potentials averaged over 5 runs are listed in Table S1. Established calibrations were then used for measuring zeta potentials of a variety of samples (e.g. liposomes, exosomes, and DNA modified magnetic particles) using TRPS. The calibration particles are initially measured at 3 applied voltages that are dependent on the applied stretch and measured baseline current. Typical baseline currents are 85 nA, 105 nA and 145 nA for voltages V1, V2, V3 respectively. Then follows a measurement of the calibration particles at at least two applied pressures and the highest voltage of the previous calibration step (V3). Each sample measurement was completed at a current of approximately 140–150 nA. For a stable and reproducible zeta potential measurement, it was imperative that the stretch of the nanopore stayed unchanged during a sample or calibration measurement of a particular dataset. Sample measurements were all completed at V2 or V3. In between sample runs the top fluid cell was washed thoroughly with electrolyte, in order to avoid cross contamination.

For each calibration step at least 300 particles were measured with typical particle concentrations of 10^9^–10^10^ particles/ml.

### Phase analysis light scattering (PALS)

Zeta potentials of polystyrene standards, exosomes and liposome were measured on a Malvern Zetasizer Nano ZS. PALS analysis was used to determine the average zeta potential of the different nanoparticle types dispersed in PBS electrolyte. Data were obtained from 15 measurement cycles and repeated three times. A fast measurement process (fast field reversal mode: FFR) was selected, because the ionic strength of the dispersing media (electrolyte) was high, in order to prolong the life of the measurement cell.

### Streaming potential measurement

Zeta potentials of thermoplastic polyurethane (TPU) membranes were measured via streaming potential and current measurements using a Surpass Instrument (Anton Paar GMBH, USA). The streaming potential of TPU was measured for a range of applied pressures within a cyclic pressure sweep, followed by the evaluation of the TPU zeta potential, applying the Helmholtz-Smoluchowski equation^61^.

## Electronic supplementary material

Supplementary Information
