# Supplementary material for: High-Resolution Single Particle Zeta Potential Characterisation of Biological Nanoparticles using Tunable Resistive Pulse Sensing
Source: Sci Rep. 2017 Dec 12;7:17479. doi: 10.1038/s41598-017-14981-x (PMC5727177; doi:10.1038/s41598-017-14981-x)
Supplement: Supplementary file 1 — Supplementary Information [file 41598_2017_14981_MOESM1_ESM.pdf]

High-Resolution Single Particle Zeta Potential Characterisation of Biological Nanoparticles using Tunable Resistive Pulse Sensing

Robert Vogel, Anoop K. Pal, Siddharth Jambhrunkar, Pragnesh Patel, Sachin S. Thakur, Eduardo Reátegui, Harendra S. Parekh, Paula Saá, Adonis Stassinopoulos and Murray F. Broom

Supplementary Info

1. **Methodology**

The calibration process is two step, the first being elctrokinetic, and the second convective. The first step has been described in detail by Blundell et al.,[1,2] but its steps are listed here again. The complete calibration process can be summarised as follows:

$1/T_x$ is averaged over at least 300 calibration particles for x = 0.30, 0.35, 0.40, 0.45, 0.50, 0.55, 0.60. $1/T^i_x$ and the time averaged total particle velocity, $v^i_{x_{tot}}$ of particle, *i*, have a proportional relationship (A1). L_x is the position within the pore that is reached after $t = T^i_x$.

$$v^i_{x_{tot}} = \frac{l_x}{T^i_x} = \frac{\int_0^{T^i_x} v^i(t)dt}{T^i_x} \tag{A.1}$$

$$\frac{1}{Tx} = \sum_{i=1}^{N} \frac{\frac{1}{T^i_x}}{N} \tag{A.2}$$

From (A1) and (A.2) follows that $v_{x_{tot}}$ is

$$v_{x_{tot}} = \sum_{i=1}^{N} v^i_{x_{tot}}/\mathrm{N} \tag{A.3}$$

For the purpose of simplicity the measured $1/T_x$ is denoted as $v_{x_{tot}}$, which is the velocity averaged over time and particles. This total particle velocity is the sum of electrokinetic and convective velocities:

$$v_{x_{tot}} = v_{x_{el}} + v_{x_{con}} \tag{A.4}$$

Equation A3 is valid for single particle velocities and also velocities averaged over many particles. The electrokinetic (electroosmotic and electrophoretic) velocity, $v_{x_{el}}$ of the calibration particles is calculated from the slope v^V_x of the linear $\frac{1}{Tx}$ vs voltage curves (Figure 1b).

$$v^{V}_{x\,Cal} = \partial v_{x_{el}} / \partial V \tag{A.5}$$

The derivative form of the electrokinetic mobility[3] (sum of electroosmotic and electrophoretic mobilities) is given in (A.5).

The convective velocity per unit pressure of the calibration particles for each T_x is calculated from the slope v^{P}_{x} of the linear $\frac{1}{Tx}$ ($\triangleq v_{x_{tot}}$) vs pressure curves. (Figure 1c).

$$v^{P}_{x\,Cal} = \partial v_{x_{con}} / \partial P \tag{A.6}$$

$v^{V}_{x\,Cal}$ and $v^{P}_{x\,Cal}$ are calculated by averaging typically more than 300 calibration particles.

Electrokinetic particle velocities of sample and calibration and respective zeta potentials are linearly related through the Smoluchowski approximation,[4] which is a very good approximation in most scenarios, where the particle size is significantly larger than the debye length.

$$\frac{(v^{i}_{x})_{el\,Sample}}{(v_{x})_{el\,Cal}} = \frac{\xi^{i}_{x\,net\,Sample}}{\xi_{net\,Cal}} \tag{A.7}$$

$(v^{i}_{x})_{el\,Sample}$ is the time averaged electrokinetic velocity of sample particle, i, at position L_x within the pore. $(v^{i}_{x})_{\,Sample}$ is the sum of time averaged convective and electrokinetic velocities of particle, *i*.

$$v^{i}_{x_{Sample}} = \frac{L_x}{T^{i}_{x}} = \frac{\int_0^{T^{i}_{x}} v^{i}(t)dt}{T^{i}_{x}} \tag{A.8}$$

The net zeta potential is the difference between particle (sample and calibration) zeta potential and the membrane zeta potential, ξ_m.

$$\xi_{p\,Sample} = \xi_{net\,Sample} + \xi_m \tag{A.9}$$

Finally the zeta potential of each sample particle, *i*, is calculated from the ratio of sample and calibration electrokinetic velocities:

$$\xi^{i}_{Sample} = \frac{\Sigma_x \xi^{i}_{x\,Sample}}{\Sigma_x} = \frac{\Sigma_x (v^{i}_{x\,Sample} - (v^{P}_{x\,Cal} * P_{diff} + inter_{x\,Cal})) / (v^{V}_{x\,Cal} * V)}{\Sigma_x} * \xi_{net\,Cal} + \xi_m \quad (A.10)$$

with V, P_{diff} and $inter_{xCal}$ being the voltage used to run the sample, the difference of applied pressures between sample and calibration runs, and the intercept of the $1/T_x$ vs voltage linear curves, respectively.

The zeta potentials of polystyrene standards and the thermoplastic polyurethane membrane for specific electrolytes were measured with PALS and streaming potential techniques (see Table S1).

2. Calibration

The zeta potential of various sized CPCs were evaluated with PALS, which is a very reproducible and accurate measurement method for samples that are uniform in zeta potential. Results from 5 runs for each particle type are listed in Table S1.

Table S1: Diameters and zeta potential of standards in PBS as determined by TRPS and PALS respectively.

Particles	Diameter [nm]	Zeta potential [mV]
CPC70	70	-18 ± 3
CPC100	114	-21 ± 2
CPC200	212	-20 ± 3
CPC350	350	-27 ± 4
CPC400	400	-36 ± 1

3. Zeta potential reproducibility using CPN and CPC particles

Zeta potential reproducibility was tested using CPN100/200 and CPC100/200 (calibration), measured with 5 different membranes on several days. Mean zeta potentials and uncertainties are listed in Table S2 and respective results shown in Figure S1.

Table S2: Zeta potential reproducibility. Zeta potentials were averaged over 5 membranes and several days.

	**Mean [mV]**	**Stdev [mV]**	**CV [%]**	**Outlier**
CPC100	-20.5	0.7	3.3	-19.1
CPC200	-19.7	0.9	4.5	-20.9
CPN100	-11.4	0.9	8.2	-12.6
CPN200	-13.2	0.8	6.1	-14.8

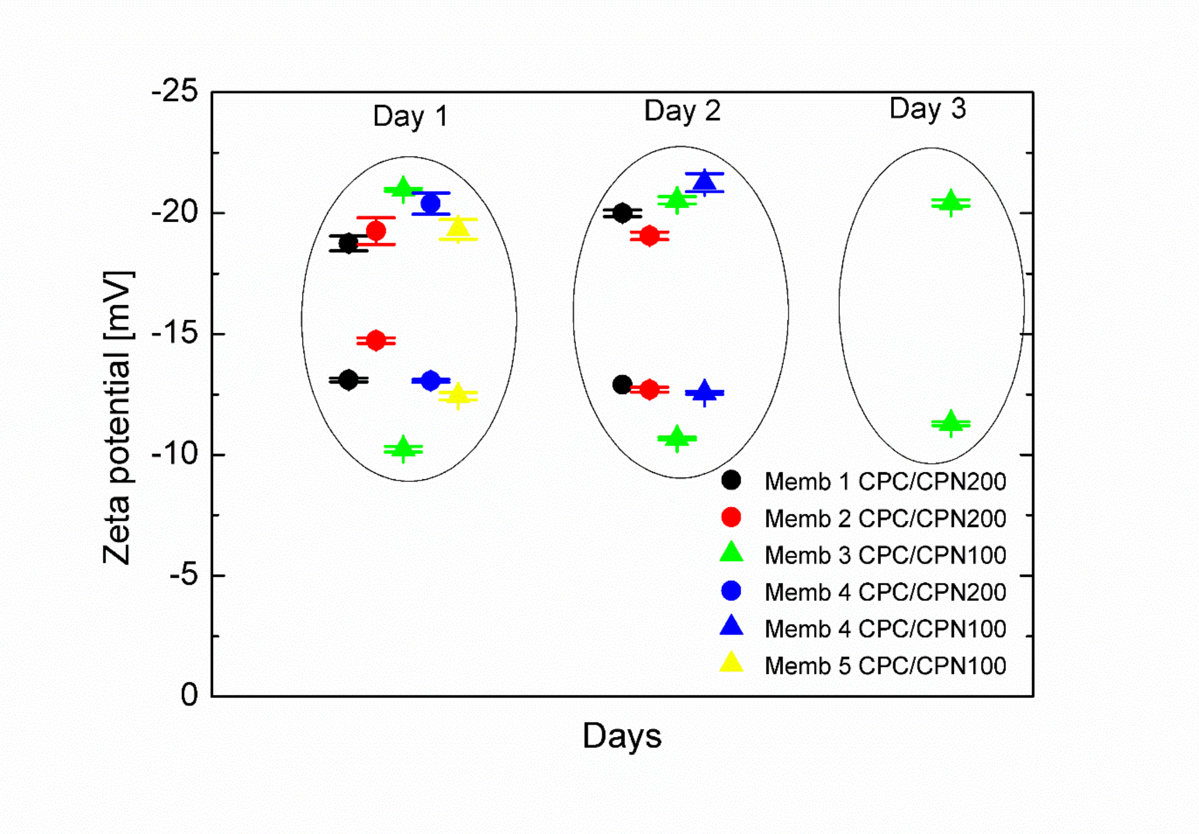

Figure S1: Zeta potential reproducibility of mixes of CPC200/CPN200 and CPC100/CPN100 tested with 5 different pores over 3 consecutive days.

Zeta potential uncertainties for CPCs lie within 5%, whilst uncertainties for CPNs are slightly higher. Please note that zeta potential CVs for CPC200 and CPN200 were 1.6% and 0.9% respectively, when samples were measured in direct succession on the same pore.

4. Effect of pore coating on zeta potential

The zeta potentials of highly and weakly charged nanoparticles were measured in the absence and presence of Izon pore coating solution to elucidate the effects of this reagent on zeta potential measurements. For this study carboxylated polystyrene CPC200, CPC350 particles and bare polystyrene CPN200 were used. The surface charge of these three different particle types were analysed with TRPS, both individually and as mix. For the mix particle concentrations needed to be adjusted to account for the lower electrophoretic mobility and hence blockade rate of the weakly CPN200. Figure S2 shows the results of the trimodal mix, recorded before and after coating. Averaged results and standard deviations from three different pore settings are displayed in Table S3.

Table S3: Zeta potential of CPN200, CPC200 and CPC350 at three different pore and calibration settings, before and after coating.

Particles	Uncoated	Coated
CPN200	-13.3 ± 0.6 mV	-12.3 ± 1.3 mV
CPC200	-20.8 ± 1.9 mV	-21.1 ± 0.9 mV
CPC350	-28.7 ± 0.7 mV	-27.2 ± 1.2 mV

The zeta potentials for CPN200, CPC350 and CPN200 before coating are in very good agreement with respective zeta potentials after coating. The small differences are within the measurement uncertainty. This demonstrates that pore coating and use of 2% ICS in the electrolyte for calibration and sample runs does not modify the surface of the translocating

particles in any measurable way. If particles were to be coated in ICS you would expect to measure a significantly reduced difference between zeta potentials of CPN and CPC, which is not the case.

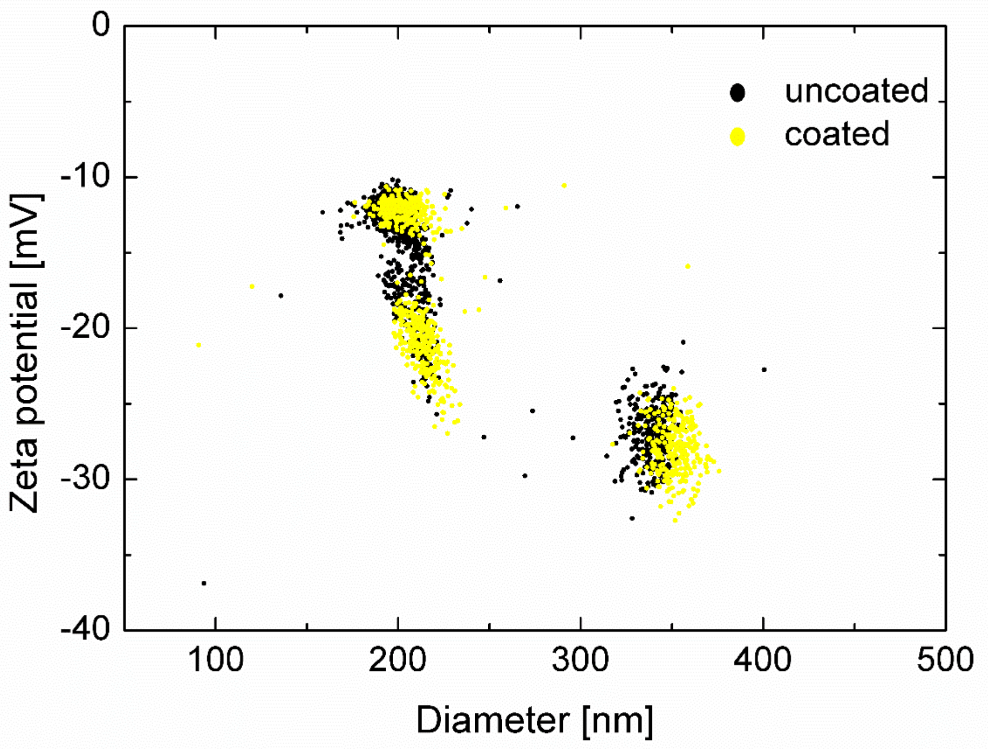

Figure S2: Zeta potential of a trimodal particle mixture of CPC200, CPN200 and CPC350 with (black) and without pore coating (yellow).

5. Comparison of PALS and TRPS zeta potential measurements of multimodal samples

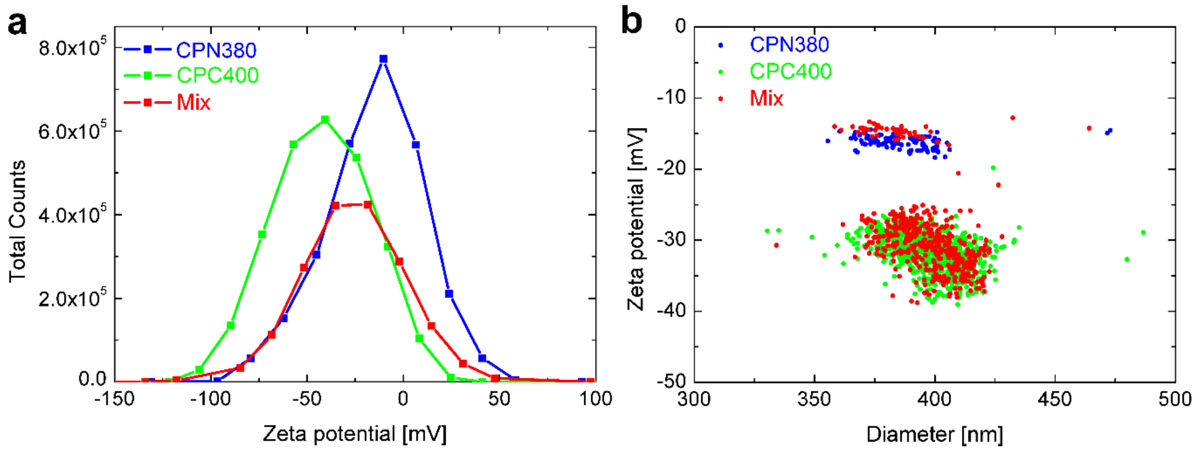

Figure S3: Comparison of phase analysis light scattering (PALS) analysis (a) and TRPS (b) of bimodal charged samples in phosphate buffered saline.[5] Analysis of the two samples of 380 nm low charged polystyrene particles and 400 nm highly charged carboxylated polystyrene particles (TRPS data points: blue for CPN380, green for CPC400; traces as labelled for PALS data) agree well between the two techniques. However, there is a huge discrepancy between these techniques when the two particle types are mixed to give a bimodal sample. Whilst TRPS can resolve the two particle types (red data points) with zeta potentials agreeing well with values from unmixed samples, PALS can only measure a solution-averaged zeta potential value (red trace).

6. Concentration and size distributions of EVs

Table S4: Concentrations $C_{80\text{-}1000}$ for a set of untreated and INTERCEPT treated samples from 6 donors. No statistically significant difference was found between samples before and after treatment.

Donors	$C_{80\text{-}1000}$ [particles/ml] **before** INTERCEPT treatment	$C_{80\text{-}1000}$ [particles/ml] **after** INTERCEPT treatment
1	$8.9*10^{10}$	$1.10*10^{11}$
2	$4.0*10^{10}$	$4.4*10^{10}$
3	$3.7*10^{10}$	$4.0*10^{10}$
4	$1.9*10^{10}$	$1.8*10^{10}$
5	$1.1*10^{10}$	$1.2*10^{10}$
6	$6.8*10^{9}$	$6.1*10^{9}$

References:

1 Blundell, E., Vogel, R. & Platt, M. Particle-by-Particle Charge Analysis of DNA-Modified Nanoparticles Using Tunable Resistive Pulse Sensing. *Langmuir* **32**, 1082-1090, doi:10.1021/acs.langmuir.5b03024 (2016).

2 Blundell, E., Vogel, R. & Platt, M. Determination of Zeta Potential via Nanoparticle Translocation Velocities through a Tunable Nanopore: Using DNA-modified Particles as an Example. *Jove-Journal of Visualized Experiments*, doi:10.3791/54577 (2016).

3 Arjmandi, N., Van Roy, W., Lagae, L. & Borghs, G. Measuring the Electric Charge and Zeta Potential of Nanometer-Sized Objects Using Pyramidal-Shaped Nanopores. *Analytical Chemistry* **84**, 8490-8496, doi:10.1021/ac300705z (2012).

4 Hunter, R. J. *Zeta Potential in Colloid Science: Principles and Applications*. (Academic Press, 1981).

5 in *Nanomedicines: Design, Delivery and Detection RSC Drug Discovery Series* (ed M. Braddock) 252-279 (2016).
